# Progress on application of spatial epidemiology in ophthalmology

**DOI:** 10.3389/fpubh.2022.936715

**Published:** 2022-08-10

**Authors:** Cong Li, Kang Chen, Kaibo Yang, Jiaxin Li, Yifan Zhong, Honghua Yu, Yajun Yang, Xiaohong Yang, Lei Liu

**Affiliations:** ^1^Department of Ophthalmology, Guangdong Eye Institute, Guangdong Provincial People's Hospital, Guangdong Academy of Medical Sciences, Guangzhou, China; ^2^Department of Ophthalmology, The First Affiliated Hospital of China Medical University, Shenyang, China; ^3^Department of Graduate, China Medical University, Shenyang, China; ^4^Department of Cataract, Baotou Chaoju Eye Hospital, Baotou, China; ^5^Department of Ophthalmology, Jincheng People's Hospital, Jincheng, China

**Keywords:** ocular disease, spatial epidemiology, risk factors, spatial statistics, disease mapping

## Abstract

Most ocular diseases observed with cataract, chlamydia trachomatis, diabetic retinopathy, and uveitis, have their associations with environmental exposures, lifestyle, and habits, making their distribution has certain temporal and spatial features based essentially on epidemiology. Spatial epidemiology focuses on the use of geographic information systems (GIS), global navigation satellite systems (GNSS), and spatial analysis to map spatial distribution as well as change the tendency of diseases and investigate the health services status of populations. Recently, the spatial epidemic approach has been applied in the field of ophthalmology, which provides many valuable key messages on ocular disease prevention and control. This work briefly reviewed the context of spatial epidemiology and summarized its progress in the analysis of spatiotemporal distribution, non-monitoring area data estimation, influencing factors of ocular diseases, and allocation and utilization of eye health resources, to provide references for its application in the prevention and control of ocular diseases in the future.

## Introduction

According to the first World Report on Vision issued by the World Health Organization in 2019, it is estimated that ~2.2 billion people suffer from vision impairment or blindness worldwide (1). Notably, this number will be still increased due to the growth of the global population (2). Therefore, preventing and controlling the occurrence and development of eye diseases is of great significance to protect people's visual health. Traditional epidemiology researches provide etiology evidence for disease control, but it does not provide sufficient variable outcomes such as spatial distribution and temporal distribution visualization.

Spatial epidemiology plays a crucial role in the analysis of spatiotemporal distribution, non-monitoring area data estimation, exploring the influencing factors of ocular diseases in specific regions, and providing strategies and measures for ocular disease prevention and control (3). Several ocular diseases have the characteristics of local incidence, and their presence and incidence are closely related to factors such as economy, population density, health care status, lifestyle, and environmental exposure in various regions (4). Recently, due to the rapid development of geographic information systems (GIS) and spatial analysis science, it has become easy to access information involving environmental and socio-demographic features, and big data on eye disease distribution, further improving the accuracy of spatial information. Therefore, spatial epidemiology has been widely performed in ocular disease prevention and control (5). This mini-review summarized the current progress of spatial epidemiology applied in the field of ophthalmology, particularly in disease prevention and control, and provided perspectives for its further application in this field.

## Search strategy and selection criteria

The literature selected for this review was sourced from Embase, PubMed/MEDLINE, Cumulative Index of Nursing and Allied Health Literature (CINAHL), Cochrane Library, and Web of Science databases. The keywords used in the search were (“spatial epidemiology” OR “spatial distribution” OR “spatiotemporal epidemiology”) AND (“ophthalmology” OR “ocular disease”). No language restrictions were applied. Eligible studies had to meet the following criteria: geospatial techniques were used in the analysis, and articles had to provide epidemic information on spatial distribution characteristics, spatial and temporal trends, or influencing factors of ocular diseases.

Searches were performed independently by both CL and LL, where after results were compared and discussed. Multiple publications of the same study were compared and the study most updated or complete was retained. In case of disagreement, XHY was consulted for a final decision.

## Overview of spatial epidemiology

Spatial epidemiology, which belongs to a branch of epidemiology, aims to descript and analyze geographic variations in disease with respect to demographic, environmental, socioeconomic, inherent, and influencing factors by using the geographic information system and other spatial technologies (6). Specifically, spatial epidemiology is concerned with the description and examination of disease and its geographic variations. The focusing issues of spatial epidemiology can be summarized as following: (i) cluster epidemiology; (ii) complete spatial randomness; (iii) geographic information system; (iv) geographic information science; (v) modifiable Areal Unit Problem; (vi) spatial analysis; and (vii) spatial autocorrelation. In terms of analysis method, spatial epidemiology relies on spatial analysis technology based on computer and information technology. It can complete the spatial data collection, analysis, exploration, and visualization (mapping) of health, disease, and health service events, and directly complete the electronic management of disease, and health service information including consultation, inquiry, and calculation (7). The massive storage and analysis capabilities of disease make data analysis more accurate, and it is possible to discover information that cannot be found by traditional epidemiology from a big data and multi-dimensional perspective. The development of spatial analysis technology will be the key to the development of spatial epidemiology, and the development of the discipline must be a process from qualitative to quantitative. Spatial statistical analysis can elevate the qualitative analysis of data to the level of quantitative description, directly measure the spatial statistical correlation between various disease data, and understand diseases from the perspective of spatial morphology, spatial location, spatial topological relationship, and spatial multidimensional dynamics (8–10). Spatial epidemiology is inseparable from spatial statistical techniques. Disease data marked by geospatial characteristics, advanced computer technology, GIS technology, and statistical technology make it possible to study disease risk on a small scale with spatial data variables. In recent years, with the rapid development of information-related modern technology, improvement of accessibility to health-related services, natural environment, social and economic big data, etc., spatial epidemiology has made considerable progress in both theory and practice and plays a more important role in the public health of ophthalmology.

## Advantages in spatial epidemiology

(i) Spatial epidemic analysis gives a spatio-temporal visualization of epidemiological data, applies spatial positioning and map visualization to visualize the spatial variation or spatiotemporal variation of disease description on the maps, and provides clues for further etiological research.
(ii) Spatial epidemiology offers a mean to extract spatial data and quantitative analysis, and it can extract real-time dynamic remote sensing image data related to diseases, such as physical geography, climate, socioeconomic and demographic information, to achieve superimposed analysis, buffer analysis, topological relationship analysis, cluster analysis and quantify influencing factors for disease pandemic.
(iii) Spatial epidemiology integrates the technical advantages of different disciplines to solve epidemiological problems, such as environmental science, ecology, and econometrics. For example, it can integrate genetics and molecular biology to study the geographical distribution of pathogen lineages.
(iv) Spatial epidemiology is demonstrated to be a feasible and advantageous method in spatial data storage, update, query, analysis, and visualization by GIS, and it can establish disease monitoring, early warning, and auxiliary decision-making system, and improve the efficiency of public health data integration.

## Main procedure in spatial epidemiology

**Figure 1:**
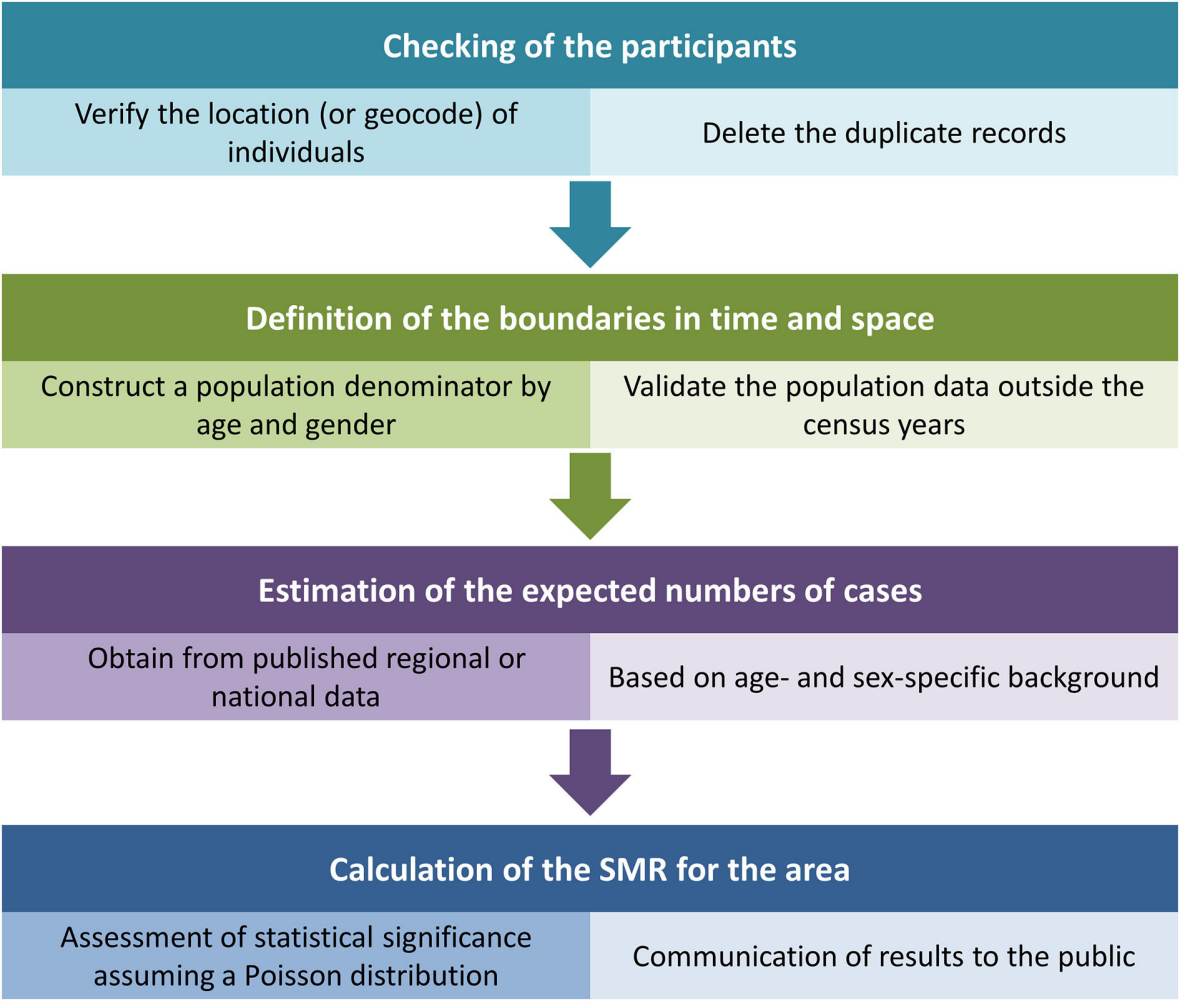
The process of the initial assessment of the data in spatial epidemiology.

Spatial epidemiology data can be divided into three main types as follows, mapping data, imaging data, and project data (11, 12). Usually, the process of the initial assessment of the data was shown in Figure 1 (6). A map consists of points, lines, and areas, and these points, lines, and areas can be defined by positional and non-positional properties in spatial reference coordinates. The map data collected by spatial epidemiology often include administrative division maps, population distribution maps, meteorological maps, or other vector maps of environmental factors (water system distribution maps, land use maps, and soil type maps). Image data are commonly from multiple sources such as aerial photography, remote sensing pictures, and digital photography. Project data are mainly from various special censuses, sampling surveys, monitoring, and hospitals.

Moreover, spatial epidemiology in the field of healthcare can be commonly divided into three main areas, including disease mapping, geographic correlation studies, and clustering of disease clusters as well as surveillance (6). Disease mapping can provide information about individualized disease clusters, quickly visualize complex geographic information, and present data that is difficult to display in traditional tables. They are applied to disease descriptions, to propose etiological hypotheses, highlight high-risk areas, and aid in policy development and resource allocation. Furthermore, disease mapping puts the results of specific disease clusters and point-source studies into an appropriate context. Geographic correlation studies examine geographic variations across population groups in exposure to environmental variables, socioeconomic and demographic measures, or lifestyle factors in relation to health outcomes measured on a geographic scale. Investigations of disease clusters and surveillance near point sources typically assume that the background risk surface is flat, against a peak of contamination sources being tested. If the background surface is uneven, there are peaks and troughs of the at-risk surface, which indicates a generalized or broad clustering of the disease.

## Application in ophthalmology

A summary of several reports implementing different spatial analyses to explore the spatial epidemiology characteristics of ocular diseases is included in Table 1.

**Table 1:** Methods for spatial analysis with examples of applications and findings in ophthalmology.

**Authors**	**Geographic unit and location**	**Years**	**Data resource**	**Geospatial techniques**	**Ocular disease**	**Key findings**
Azzam et al. (13)	United States	2004–2019	DED-related queries estimating users' intent	Internet epidemiological tools along with geographic information system data from the environment as a mapping technique plus Multivariable regression models	Dry eye disease	Urban living and seasonality were stronger risk factors of dry eye disease searches than temperature, humidity, sunshine, pollution, or region
Um et al. (14)	Republic of Korea	2010–2012	The 5th Korea National Health and Nutrition Examination Survey	Serial multiple logistic regression plus ArcGIS	Dry eye disease	The prevalence of dry eye disease was higher in south Korea, which can be influenced by the degree of urbanization and environmental factors such as humidity and sunshine duration
Yohannan et al. (15)	Tanzania	NA	Partnership for the Rapid Elimination of Trachoma (PRET) Trial	Global positioning system plus A Galaxy Tab 2.0 7-inch Android device	Chlamydia trachomatis infection and active trachoma	Chlamydia trachomatis infection clusters after multiple rounds of mass treatment with azithromycin
Broman et al. (16)	Tanzania	NA	Pilot survey on trachoma	Global positioning system plus A k-function analysis	Ocular chlamydial infection	Ocular chlamydia spreads between households with children or that nearby households share the same risk factors for infection
Wong et al. (17)	Hong Kong	2000/2001 to 2016/2017	The annual health checks conducted at Student Health Service Centers	Spatial autocorrelation	Visual impairment	The difference in prevalence of reduced visual acuity between Hong Kong and mainland China has decreased in recent years. Cross-border students living in mainland China were associated with a lower risk for reduced visual acuity
Virgili et al. (18)	16 European countries	1983–1994	The European Cancer Registry-based study	Multilevel Poisson regression	Uveal melanoma	Standardized incidence rates increased from south to north across registries, from a minimum of <2 per million in registries of Spain and southern Italy up to >8 per million in Norway and Denmark
Culham et al. (19)	United Kingdom	1997–1998	A telephone questionnaire	Geographic information system	Visual impairment	The distribution of low vision consultations was geographically uneven and there appears to be scarcity in some areas
Kozioł et al. (20)	Poland	2017	The National Health Fund database	Moran's I statistics and Spatial Autoregressive	Diabetic retinopathy	The analyses of social, demographic, and systemic factors co-existing with DR revealed that level of income and access to ophthalmologic and diabetic services are crucial in DR prevalence in Poland
Wu et al. (21)	China	2013–2017	Cataract Revision Surgery Information Reporting System from 2013–2017	ArcGIS10.0 software plus spatial autocorrelation analysis plus SaTScan 9.5 software	Cataract surgery	Cataract surgery rates in China showed increasing trend year by year and were randomly distributed, with spatial clustering, and Anhui was reported as a low-high clustering region
Yang et al. (22)	China	2016–2019	Environmental Health and Myopia Prevention and Control Project	Normalized difference vegetation index	Myopia	There is a negative association between green space exposure and myopia
Dadvand et al. (23)	Spain	2012–2015	The BRain dEvelopment and Air polluTion ultrafine particles in scHool childrEn (BREATHE) project	Land-use regression models	Use of spectacles	There is an increased risk of spectacles use associated with exposure to traffic-related air pollution
Chung et al. (24)	Taiwan	2012	The Taiwan Biobank	Hybrid kriging/land-use regression model	Dry eye syndrome	Significant associations of ambient NO_2_ concentration, relative humidity and temperature with dry eye syndrome indicated the importance of increased environmental protection in the female population
Chua et al. (25)	United Kingdom	2006–2010	UK Biobank	Land use regression models	Cataract surgery	There was a 5% increased risk of future cataract surgery associated with an exposure to PM_2.5_, NO_2_, and Nox
Shah et al. (26)	Canada	2013–2014 and 2016	Canadian Community Health Survey	Cross-classification mapping	Optometry services	A nationwide overview of vision care provided by optometrists identifying gaps in geographic availability relative to “supply” and “need” factors
Vu et al. (27)	United States	2018–2019	American Glaucoma Society and American Association for Pediatric Ophthalmology and Strabismus	ArcGIS Pro (Esri)	Primary congenital glaucoma care	Approximately 14–94 new primary congenital glaucoma cases/year may be at risk of delayed diagnosis as a result of living in a potential service desert
Tan et al. (28)	China	2006–2017	The largest database of uveitis cases	Choropleth maps	Uveitis	A 10 μg/m^3^ increase in PM_2.5_ concentration was associated with a one-case per 10 individuals increase in uveitis onset
Tan et al. (29)	China	2006–2017	The largest database of uveitis cases	Choropleth maps	Uveitis	Rising temperature can affect large-scale uveitis onset

*NA, not applicable*.

### Analysis of the spatial and temporal distribution of ocular diseases

Disease reports, sentinel surveillance, and epidemiological investigations on several eye health such as visual impairments, corneal lesions, cataracts, and occupational eye disease are mostly presented in the spatial form of administrative divisions or surveillance locations. GIS can map eye disease cases and their influencing factors and reveal the correlation between them (30). Furthermore, the thematic map intuitively displays the locations where the distribution of eye diseases is relatively concentrated, demonstrates temporal and spatial dynamic changes of eye diseases, and reveals the hidden spatial information in the chronic epidemic process.

### Exploring the spatial distribution characteristics of ocular diseases

Spatial distribution characteristics refer to the global, national or local scale spatial correlation. Global scale spatial correlation is often measured by the entire Moran's *I* value or high/low clustering to measure whether there is an agglomeration of ocular diseases in the study area. For local spatial correlation, it is determined by the local Moran's *I* value or hot spot analysis to evaluate the correlation of ocular disease distribution in a spatial unit, and even determine the aggregation area. The location of providers plays an essential role in planning an accessible service. The distribution of services across the United Kingdom was mapped using the GIS and showed that low vision consultations were unevenly distributed across the country. Specifically, the scarcity of services was in coastal and rural areas where the proportion of the population living there with visual impairments was high (19). Kozioł et al. used GIS with spatial analytical methods to determine the distribution of diabetic retinopathy (DR) prevalence and suggested that there were significant differences in the proportion of DR with a score of the Moran Index of 0.18 (*P* < 0.01). Specifically, the high prevalence of DR was concentrated in the southwest part of Poland but the low prevalence was particularly located in the center of the Country in the years 2013–2017 (20). In addition, Wu et al. established that GIS uses its spatial analysis to show spatial clustering of cataract surgeries in China, with Anhui province being a low-high clustering region. Furthermore, spatial autocorrelation analysis indicated that the distribution status of cataract surgery rates (CSR) in different regions of China also showed some spatial heterogeneity (21). The spatial map clearly demonstrated the high-risk areas with low CSR in China and provided a reference for the government or blindness prevention organizations to conduct targeted prevention and control measures to strengthen the prevention and treatment of cataract blindness.

### Analysis of spatial and temporal trends of ocular diseases

In spatial epidemiology, spatial and temporal trends of disease can be dynamically described by the SaTScan software. In order to compensate for the shortcoming of spatial autocorrelation analysis lacking the size and extent of agglomeration, Wu et al. applied staged spatial and temporal scan analysis to registered cases of cataract surgery in 2013–2017 in China and found there were 18 areas of aggregation in two stages (2013–2015 and 2016–2017), and statistically significant differences in each area of aggregation with a particularly clear scope of aggregation (21). From 2013 to 2017, a gradual decrease in area aggregation was observed in the staged spatiotemporal scans, which showed cataract surgery prevention and control work has made some progress in China (21).

### Explore the influencing factors of ocular disease

The geographical detector model was widely used to identify the main factors driving the spatial-temporal variations of disease. In methodology, it is used to measure the consistency of the spatial distribution pattern of the dependent variable and the independent variable, that is, the correlation between exposure and outcomes by the Pearson correlation coefficient or nonparametric test (31). Yang et al. used normalized difference vegetation index (NDVI) as a measure of green space exposure, which was defined as the ratio of the difference between near-infrared reflectance and red visible reflectance to their sum, which ranges between −1 and 1, with a higher value indicating more greenness (22). They found that there was a negative association between green space exposure and myopia, giving a guide for the development of prevention strategies targeting the onset of myopia.

The Land-use Regression (LUR) model is one of the commonly used methods for simulating the spatial and temporal differentiation of urban-scale air pollution. With LUR, statistical methods use a range of GIS–derived predictor variables, such as traffic intensity, population, topography, and land use, to offer fit-for-purpose predictions on long-term concentration at a fine spatial scale (32, 33). Particularly, researchers combine LUR with ground-based measurements, and satellite remote sensing (SRS) to generate high-resolution pollution maps for air pollutants (34). Notably, LUR models consider the land use conditions and can improve accuracy in predicting the variation of various air pollutants with high explanatory power (*R*^2^ > 0.85) (35). For example, Dadvand et al. (23) established the LUR model to predict nitrogen dioxide (NO_2_) and ambient fine particulate matter (PM_2.5_) light absorbance at home, and further found there was an increased risk of spectacles use with exposure to traffic-related air pollution. In order to estimate ground-level concentrations of multiple air pollutants such as PM_2.5_, sulfur dioxide (SO_2_), ozone (O_3_), and NO_2_ based on the municipality, a hybrid kriging/LUR model was used, and the results indicated female exposure to high levels of NO_2_ had significantly increased risk of dry eye syndrome (24). In a recent prospective, observational study to examine the relationship between ambient air pollution exposure and incident cataract surgery in the UK, those outdoor air pollution included particulates, NO_2_, and nitrogen oxides (NO_x_) was estimated based on LUR models developed by the European Study of Cohorts for Air Pollution Effects (ESCAPE) project (36), and their impacts on the risk of future cataract surgery were increased (25).

Although the LUR model explains the spatial and temporal variability levels, which are dominated by traffic, meteorology, and construction, the performance of this model is decreased in some areas around the local center. This discrepancy may be due to the excessive development gap between central and peripheral areas. Herein, the performance of the LUR model in prediction across different regions should be further improved (37).

### Explore the allocation and utilization of eye health resources

In many countries and regions, health resources, especially eye health services, are unevenly distributed. Some underdeveloped locations, particularly in rural and remote areas, do not have the same access to a range of primary eye care professionals. In some countries, although eye health resources are set up in areas serving nearby target populations, their actual utilization levels are often unbalanced. Through spatial epidemiology, it is possible to understand the real utilization of eye health resources by different groups of people, which is conducive to the optimization of resources.

Based on spatial analysis, Shah et al. evaluated the distribution of eye care health relative to population needs and utilization of vision services in Canada. Cross-classification mapping compared optometrist distribution to self-reported use of vision care services in relation to need. Eye care distribution to the use of vision care services in relation to population needs was compared by cross-classification mapping. According to this spatial analytic study, the nationwide distribution of eye care services is variable across Canada and predominantly concentrated in cities (26). Even though generalization of the findings should be cautious with data quality on health service location, utilization status, and potential geographical factors, these spatial epidemic outcomes offer a better understanding and evaluation of accessed health care resources and help policy-makers, and care providers to consider facilitating the use of eye care services at national, provincial, and health region levels.

Moreover, according to geospatial service coverage analysis involving 60 min drive time regions to providers generated by ArcGIS Pro, ~14–94 infants with primary congenital glaucoma (PCG) per year may acquire the consequences of delayed diagnosis and failure to obtain timely intervention due to living in a potential service desert (27). This study indicated that geospatial service coverage analysis may be a useful tool for identifying underserved regions for referrals and evaluation of infants with PCG and assist in targeting screening programs in low-access areas for PCG prevention.

Tan et al. (28) estimated the role of PM_2.5_ exposure on uveitis burden using choropleth maps to precisely characterize geographical variations. Additionally, their recent report visually showed spatial uveitis variations in mainland China by choropleth maps and found that there was a significant association between rising temperature and an increase in the incidence of uveitis (29).

## Summarize and challenges

In sum, with the development of GIS, global positioning system, remote sensing technology, and spatial analytic methods, some novel implementation technologies have been developed for spatial epidemiology, which has promoted spatial epidemiology widely used. In ophthalmology, spatial epidemiology has played an essential role in exploring the distribution of eye diseases, their characteristics of temporal and spatial changes, as well as the macro- and micro-influencing factors, providing more references for implementing prevention and control strategies tailored to local conditions. Notably, when using spatial epidemiology, as the scale of the investigation becomes narrowed to a particular small area or group of areas, the reduced size of the population at risk will lead to small numbers of events and unstable risk estimates. Specifically, at the broader scale, purely local variations in data quality are likely to essentially cancel out, whereas, at the small-area scale, these variations could lead to severe biases if not detected.

There are some challenges needing further improvements.

(i) Spatial epidemiology opens a new way to form a more unified paradigm by integrating epidemiology with genetics, geography, and informatics. In the future, more disciplines such as molecular biology, and genomics should be integrated with spatial epidemiology and performed in the field of clinical medicine to promote disease prevention and control.
(ii) Although there are many epidemiological databases on eye diseases, some spatial attribute data such as geocoding, socio-demographic, and behavioral information that are not related to the disease are relatively rough or lacking, making in-depth spatial analysis difficult to achieve. Thus, there is a need to ensure the comprehensiveness and accuracy of the geocoded, socio-demographic and behavioral information collected. In addition, researchers may consider adding questions such as “address code/coordinates” when designing the questionnaire.
(iii) The application of different spatial analytic methods has its own features. For example, the spatial autocorrelation method is good at identifying the location of high-prevalence clusters of eye diseases, while the SaTScan software can keenly capture areas that are currently at a low prevalence level together with a fast growth rate of cases. Furthermore, the relationship between local epidemic trends and the overall epidemic trend can be estimated by the Bayesian hierarchical model. Spatial interpolation technology incorporates geographic location which is attributed to the model for estimation. Confounders such as social-economic, cultural, environmental, population, and individual behavior characteristics are difficult to obtain comprehensively and accurately, resulting in a certain deviation between the estimated and actual value. Meanwhile, the exploration of the influencing factors on eye disease also faces the same problem, suggesting that it is necessary to further strengthen the multi-source database link and its integration with potential confounders and be beneficial to disease prevention.

## Data availability statement

The original contributions presented in the study are included in the article/supplementary material, further inquiries can be directed to the corresponding authors.

## Author contributions

All authors listed have made a substantial, direct, and intellectual contribution to the work and approved it for publication.

## Funding

This research was supported by the Science and Technology Program of Guangzhou, China (202002020049) (XY), Project of Special Research on Cardiovascular Diseases (2020XXG007) (XY), and GDPH Supporting Fund for Talent Program (LL). The funders had no role in the study design, data collection, and analysis, decision to publish, or preparation of the manuscript.

## Conflict of interest

The authors declare that the research was conducted in the absence of any commercial or financial relationships that could be construed as a potential conflict of interest.

## Publisher's note

All claims expressed in this article are solely those of the authors and do not necessarily represent those of their affiliated organizations, or those of the publisher, the editors and the reviewers. Any product that may be evaluated in this article, or claim that may be made by its manufacturer, is not guaranteed or endorsed by the publisher.
